# Supplementary material for: Missense3D-PPI: A Web Resource to Predict the Impact of Missense Variants at Protein Interfaces Using 3D Structural Data
Source: J Mol Biol. Author manuscript; Available in PMC 2025 Mar 24. (PMC7617523; doi:10.1016/j.jmb.2023.168060)
Supplement: SI-text [file EMS203879-supplement-SI_text.docx]

**Supplementary Data**

**Missense3D-PPI: a web resource to predict the impact of missense variants at protein interfaces using 3D structural data**

Cecilia Pennica ^1^, Gordon Hanna ^1^, Suhail A Islam ^1^, Michael JE Sternberg ^1^, Alessia David ^1^

^1^ Centre for Integrative Systems Biology and Bioinformatics, Department of Life Sciences, Imperial College London, London SW7 2AZ, UK

**CONTENT Page**

**Supplementary Material** *2*

**Table S1** *Structural features analysed by Missense3D-PPI*

*for residues participating in protein-protein interactions* 5

**Table S2** *Training and testing sets* 6

**Table S3** *Performance of predictors at different energy thresholds*  *7*

**Table *S4*** *P value from the McNemar test at different energy thresh*olds 7

**Figure S1** *Percent sequence identity of proteins between training*

*and test sets* 8

***Figure S2*** *Performance of the individual structural features on*

*the Missense3D-PPI training set*  *9*

**Figure S3** *Performance of the individual structural features included*

*in Missense3D-PPI* *10*

**Figure S4** *Performance of Missense3D-PPI structural features on*

*core residues (on test set)* *11*

**Figure S5** *Performance of Missense3D-PPI structural features*

*on rim residues (on test set) 12*

**Figure S6** *Damaging variants affecting protein interfaces are*

*correctly identified by Missense3D-PPI: two case studies* *13*

**Figure S7** *Sequence conservation of wild type residues harbouring*

*missense variants included in the dataset* 14

**Supplementary Material**

**Identification of homologues proteins**

The HH-suite, an open-source software for sequence similarity searches based on pairwise alignment of hidden Markov models (HMM) [1] was used to identify homologous proteins. Briefly, fasta files for all proteins in the dataset harbouring variants were collected from UniProt [2]. The program HHblits was then used to search the HHblits database (UniRef30_2021_06) with the query sequences and create multisequence aligments (MSAs). HHmake was run to create hidden Markov models (HMM) from the input MSAs. Finally, HHsearch was used to search the HHsearch database (version 3) of HMMs with the query HMMs built using HHmake.

Proteins were then distributed between the training and test set according to the E-value. Two proteins were considered homologues and placed in the same dataset (either training or test) if the E-value was < 0.001. If the E-value between two proteins was ≥ 0.001, they were allowed to be added to different data sets. The percent sequence identity of proteins between training an test sets is presented in Figure S1. The highest percent sequence identity between two proteins of the test and training sets was 27% between UniProt Ids P61960 and O43602. This corresponds to an E-value of 21 and covered a 60 amino acid long region. These two proteins were manually inspected and no common domains, according to InterPro [3], were identified.

Furthermore, we manually assessed all the remaining proteins across the training and test sets with a percent sequence identity <27%. In only two cases, the proteins in training and test sets shared the same domain according to InterPro annotation. However, the variants were not located in the shared domain, and therefore the placement of the two proteins was not modified. Next we aimed to assess whether shared superfamilies, which would be structurally similar, were present in the training and test sets by using SCOPe classification [4], however it was not possible to map all our PDBs to SCOPe superfamilies and we were only be able to map 358 out of 545 PDBs (65% of PDBs mapped in both training and test set). In only two cases, a protein in the test set shared a SCOPe superfamily domain with a protein in the training set. In these two cases, however, we found that there were no instances of a common superfamily/superfamily protein interface in which the training and testing variants occupied equivalenced residue positions.

**Mapping of variants onto 3D structures**

Missense3D-PPI uses the first biological unit of the PDB file containing the complex harbouring the interface variant. If the variant position is provided according to UniProt sequence numbering, variants are mapped to the protein structure using an in-house algorithm. The mutant structure is generated following the same protocol implemented in Missense3D [5] and described in the main text.

**Definition of evaluation metrics**

The following were used to assess the performance of Missense3D-PPI:

True positive (TP) = number of damaging variants correctly identified

True negatives (TN) = number of benign variants correctly identified

False positive (FP) = number of benign variants incorrectly identified

False negatives (FN) = number of damaging variants incorrectly identified

Sensitivity = TP / (TP + FN)

Specificity = TN / (TN + FP)

True Positive Rate (TPR) = Sensitivity

False Positive Rate (FPR) = FP / (FP+TN)

TPR/FPR ratio

Accuracy = TP+TN / (TP+TN+FP+FN)

Matthews Correlation Coefficient (MCC)

**Table S1 Structural features analysed by Missense3D-PPI for residues participating in protein-protein interactions (interface residues).**

For each feature, a brief description and the interface residue (core, rim or support) on which it is applied is presented.

| Features | Description | Interface residue |
| --- | --- | --- |
| Interface H-bond breakage/formation | The substitution breaks or forms side-chain/side-chain H-bond(s) and/or side-chain/main-chain bond(s) between two interchain residues. The maximum H-bond N–O length is 3.9Å. | Core, Support |
| Interface salt bridge breakage/formation | The substitution breaks or forms a salt bridge between two interchain residues. The maximum N–O bond length is 5.0Å. | Core, Support |
| Interface charge switched | The substitution replaces a charged interface residue with a residue of opposite charge [e.g. from arginine to aspartic acid]. | Core, Support |
| Interface charge introduced | The substitution replaces a non-charged interface residue with a charged one (histidine, lysine, arginine, glutamic acid or aspartic acid). | Core, Support |
| Interface charge replaced | The substitution replaces a charged interface residue (histidine, lysine, arginine, glutamic acid or aspartic acid) with a non-charged one. | Core, Support |
| Interface buried/exposed switch | The substitution results in a change between buried and exposed state of the target residue. (RSA < 9% for buried and the difference between RSA has to be at least 5%) | Core, Support |
| Interface hydrophilic introduced | The substitution replaces a hydrophobic interface residue with a hydrophilic one. | Core, Support, Rim |
| Interface disulfide bond breakage | The substitution breaks an interchain disulfide bond that was present in the wild-type structure. The maximum S–S length for the bond is 3.3 Å. | Core, Support, Rim |
| Interface clash | The mutant structure has a MolProbity clash score ≥30 and the increase in clash score is >18 compared to the wild type. | Core, Support, Rim |
| Interface secondary structure altered | The substitution results in a change in the DSSP secondary structure assignment. | Core, Support, Rim |
| Interface cavity altered | The substitution leads to an expansion (≥70 Å^3^) or contraction (<70 Å^3^) of the cavity volume. | Core, Support, Rim |
| Interface cis Pro replaced | The substitution replaces an interface proline, which was in cis configuration in the wild type. | Core, Support, Rim |
| Interface Gly in a bend | The substitution replaces an interface glycine, which is located in a bend curvature (reported “S” in DSSP). | Core, Support, Rim |
| Interface disallowed phi/psi | The mutant residue is in an outlier region, while the wild-type residue is in the favoured/allowed region. | Core, Support, Rim |
| Interface Pro introduced | The substitution introduces a proline at the interface. | Core, Support, Rim |
| Interface Gly/Tyr/Trp replaced | The substitution replaces an interface glycine, tyrosine, or tryptophan residue with any other residue. | Core, Support, Rim |

RSA, relative solvent accessibility

**Table S2 Training and testing sets.**

|  | **Training set** | **Testing set** |
| --- | --- | --- |
| Total variants | 640 | 639 |
| Pathogenic variants | 320 | 413 |
| Benign variants | 320 | 226 |
| Rim variants | 435 | 381 |
| Core variants | 196 | 242 |
| Support variants | 9 | 16 |
| Number of proteins | 310 | 124 |
| Number of complexes | 375 | 170 |

**Table S3 Performance of Missense3D-PPI and other variant predictors.** A ΔΔG =1.0 (S3A) and ΔΔG =2.0 (S3B) are used to define variants as damaging.

S3A - Threshold 1.0 kcal/mol

|  | **Missense3D-PPI** | **Missense3D** | **MutaBind2** | **BeAtMuSiC** | **mCSM-PPI2** |
| --- | --- | --- | --- | --- | --- |
| **MCC** | 0.28 | 0.12 | 0.24 | 0.20 | 0.17 |
| **Accuracy** | 58% | 40% | 60% | 54% | 51% |
| **Sensitivity** | 44% | 8.5% | 52% | 41% | 33% |
| **Specificity** | 84% | 98% | 71% | 77% | 83% |
| **TPR** | 0.44 | 0.08 | 0.52 | 0.41 | 0.32 |
| **FPR** | 0.16 | 0.02 | 0.28 | 0.22 | 0.17 |

S3B - Threshold 2.0 kcal/mol

|  | **Missense3D-PPI** | **Missense3D** | **MutaBind2** | **BeAtMuSiC** | **mCSM-PPI2** |
| --- | --- | --- | --- | --- | --- |
| **MCC** | 0.28 | 0.12 | 0.12 | 0.15 | 0.04 |
| **Accuracy** | 58% | 40% | 43% | 45% | 37% |
| **Sensitivity** | 44% | 8.5% | 12% | 17% | 2.5% |
| **Specificity** | 84% | 98% | 95% | 93% | 99% |
| **TPR** | 0.44 | 0.08 | 0.12 | 0.17 | 0.024 |
| **FPR** | 0.16 | 0.02 | 0.04 | 0.06 | 0.013 |

**Table S4 P-values from the McNemar test at different energy thresholds.**

| **Missense3D-PPI** | **Missense3D** | **MutaBind2** | **BeAtMuSiC** | **mCSM-PPI2** |
| --- | --- | --- | --- | --- |
| **ΔG cut-off = 1.0 kcal/mol** | 4.23x10^-16^ | 0.5 | 0.14 | 0.0002 |
| **ΔG cut-off = 1.5 kcal/mol** | 4.23x10^-16^ | 0.0025 | 3.4x10^-5^ | 1.5x10^-12^ |
| **ΔG cut-off = 2.0 1kcal/mol** | 4.23x10^-16^ | 5.9 x 10^-10^ | 8.1 x 10^-10^ | 8.6 x 10^-22^ |

**Figure S1 Percent sequence identity of proteins between training and test sets.** If the E-value between two proteins was ≥ 0.001, they were allowed to be added to different data sets.


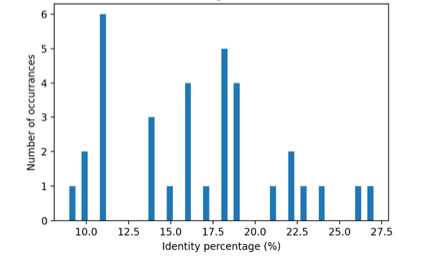


**Figure S2 Performance of the individual structural features on the Missense3D-PPI training set**

The true positive (TPR) and false positive (FPR) rates are presented as blue and grey bars, respectively. The ratios of TPR to FPR is also given and presented within boxes.


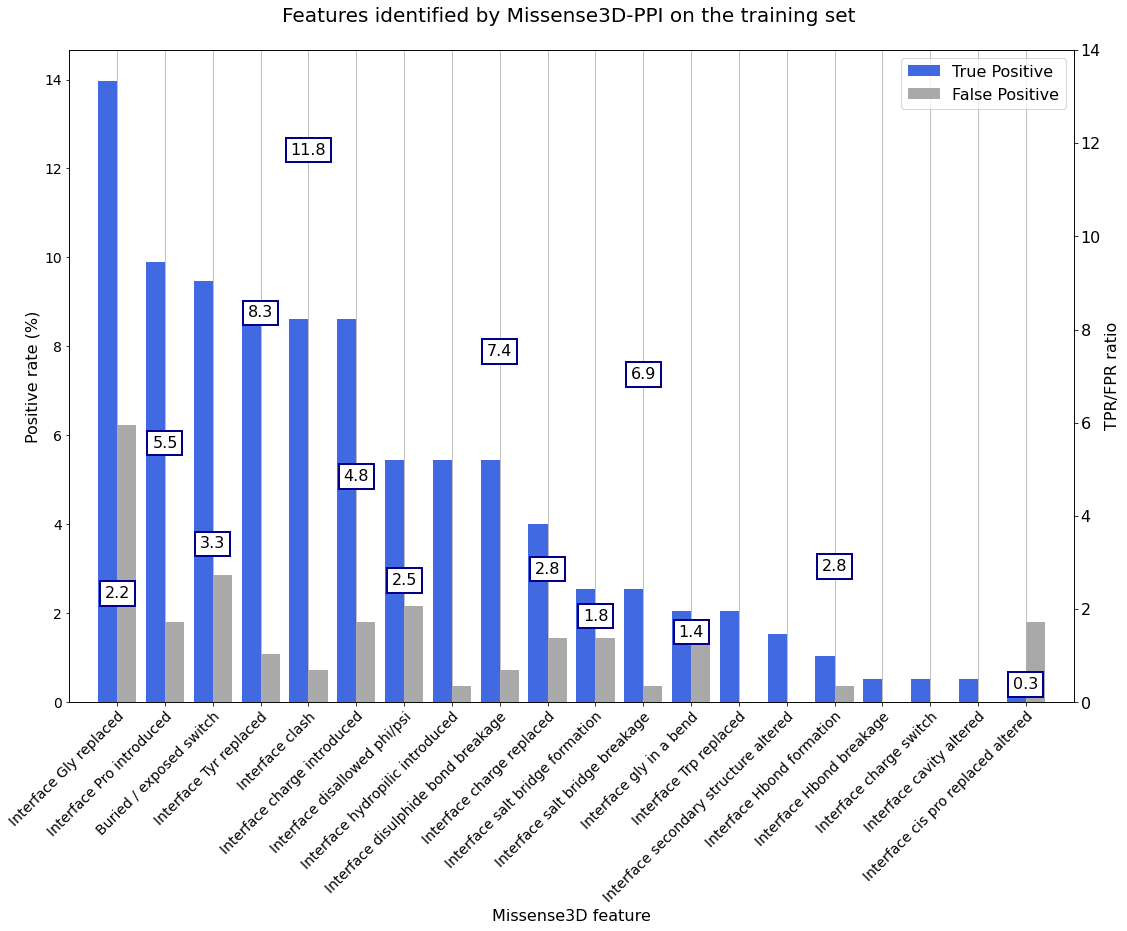


**Figure S3 Performance of the individual structural features included in Missense3D-PPI**

The true positive (TPR) and false positive (FPR) rates are presented as blue and grey bars, respectively. The ratios of TPR to FPR is also given and presented within boxes.

**
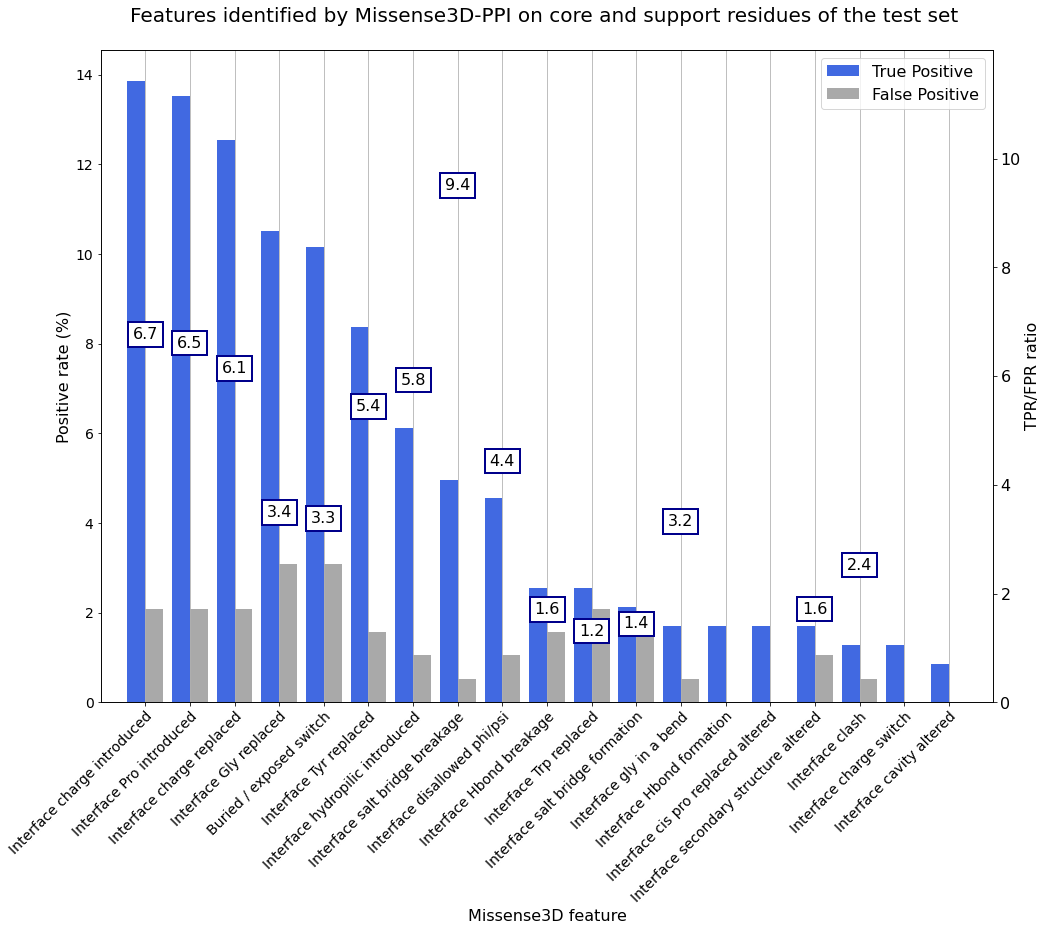
**

**Figure S4 Performance of Missense3D-PPI structural features on core residues (on test set)**

The true positive (TPR) and false positive (FPR) rates are presented as blue and grey bars, respectively. The ratios of TPR to FPR is also given and presented within boxes.

**
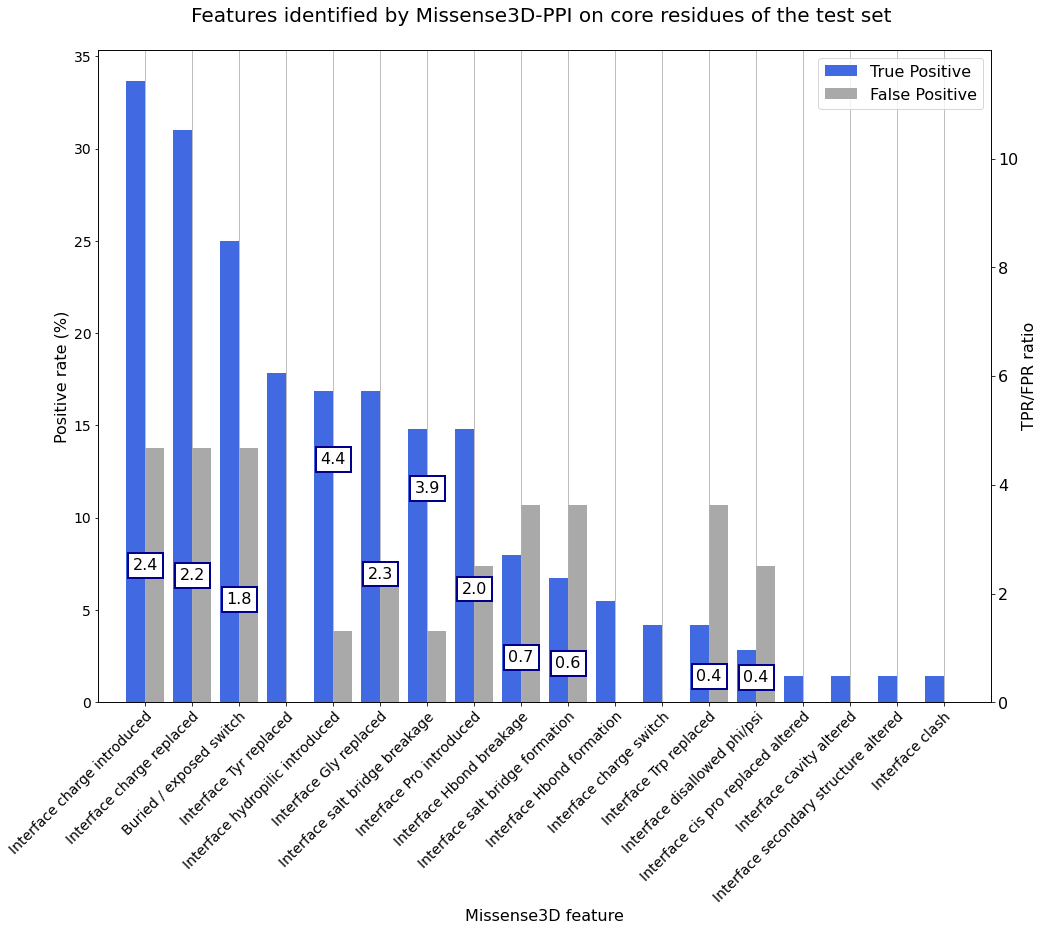
**

**Figure S5 Performance of Missense3D-PPI structural features on rim residues (on test set)**

The true positive (TPR) and false positive (FPR) rates are presented as blue and grey bars, respectively. The ratios of TPR to FPR is also given and presented within boxes.

**
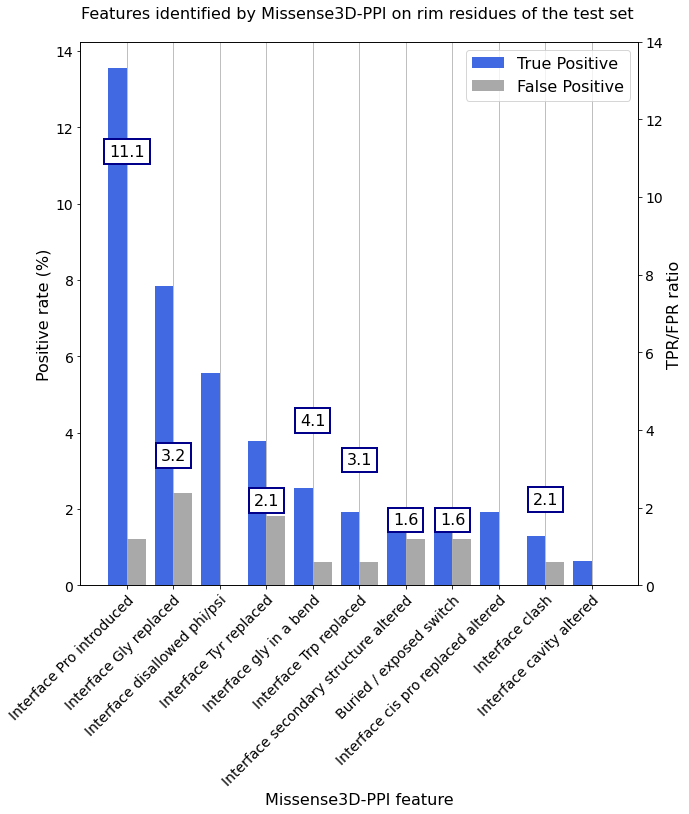
**

**Figure S6 Damaging variants affecting protein interfaces are correctly identified by Missense3D-PPI: two case studies**

Top panel, p.Ser336Arg in the human enzyme ACADM (PDB: 4p13). Bottom panel, p.Gly41Arg in the AGXT human enzyme (PDB: 4kyo). The two chains of a dimer are presented in cyan and green and the wild-type / variant residue in red and blue for positively charged residues.

**Figure S7 Sequence conservation of wild type residues harbouring missense variants included in the dataset.** Data are presented for the entire dataset and for the variants included in either the training or test set. The conservation scores of the wild type residues were obtained from [6].

**References**

[1] M. Steinegger, M. Meier, M. Mirdita, H. Vöhringer, S.J. Haunsberger, J. Söding, HH-suite3 for fast remote homology detection and deep protein annotation, BMC Bioinformatics. 20 (2019) 1–15. https://doi.org/10.1186/S12859-019-3019-7/FIGURES/7.

[2] UniProt: the universal protein knowledgebase in 2021, Nucleic Acids Res. 49 (2020) D480–D489. https://doi.org/10.1093/nar/gkaa1100.

[3] T. Paysan-Lafosse, M. Blum, S. Chuguransky, T. Grego, B.L. Pinto, G.A. Salazar, M.L. Bileschi, P. Bork, A. Bridge, L. Colwell, J. Gough, D.H. Haft, I. Letunić, A. Marchler-Bauer, H. Mi, D.A. Natale, C.A. Orengo, A.P. Pandurangan, C. Rivoire, C.J.A. Sigrist, I. Sillitoe, N. Thanki, P.D. Thomas, S.C.E. Tosatto, C.H. Wu, A. Bateman, InterPro in 2022, Nucleic Acids Res. 51 (2023) D418–D427. https://doi.org/10.1093/nar/gkac993.

[4] J.-M. Chandonia, L. Guan, S. Lin, C. Yu, N.K. Fox, S.E. Brenner, SCOPe: improvements to the structural classification of proteins - extended database to facilitate variant interpretation and machine learning, Nucleic Acids Res. 50 (2022) D553–D559. https://doi.org/10.1093/nar/gkab1054.

[5] S. Ittisoponpisan, S.A. Islam, T. Khanna, E. Alhuzimi, A. David, M.J.E. Sternberg, Can Predicted Protein 3D Structures Provide Reliable Insights into whether Missense Variants Are Disease Associated?, Journal of Molecular Biology. 431 (2019) 2197–2212. https://doi.org/10.1016/j.jmb.2019.04.009.

[6] N. Malhis, S.J.M. Jones, J. Gsponer, Improved measures for evolutionary conservation that exploit taxonomy distances, Nat Commun. 10 (2019) 1556. https://doi.org/10.1038/s41467-019-09583-2.
